# Linking Opinions Shared on Social Media About COVID-19 Public Health Measures to Adherence: Repeated Cross-Sectional Surveys of Twitter Use in Canada

**DOI:** 10.2196/51325

**Published:** 2024-08-13

**Authors:** José Denis-Robichaud, Erin E Rees, Patrick Daley, Christina Zarowsky, Assane Diouf, Bouchra R Nasri, Simon de Montigny, Hélène Carabin

**Affiliations:** 1 Groupe de recherche en épidémiologie des zoonoses et santé publique Université de Montréal Saint-Hyacinthe, QC Canada; 2 Public Health Risk Sciences Division National Microbiology Laboratory Public Health Agency of Canada Saint-Hyacinthe, QC Canada; 3 Faculté de médecine vétérinaire Université de Montréal Saint-Hyacinthe, QC Canada; 4 Policy Research Group Canadian Heritage Gatineau, QC Canada; 5 School of Public Health Université de Montréal Montreal, QC Canada; 6 Centre de Recherche en Santé Publique Université de Montréal Montreal, QC Canada; 7 Department of Infectious and Tropical Diseases Cheikh Anta Diop University Dakar Senegal; 8 Centre de recherches mathématiques Université de Montréal Montreal, QC Canada; 9 The Data Informatics Center of Epidemiology PathCheck Foundation Cambridge, MA United States

**Keywords:** adherence to mask wearing, adherence to vaccination, social media, sociodemographic characteristics, Twitter, COVID-19, survey data

## Abstract

**Background:**

The effectiveness of public health measures (PHMs) depends on population adherence. Social media were suggested as a tool to assess adherence, but representativeness and accuracy issues have been raised.

**Objective:**

The objectives of this repeated cross-sectional study were to compare self-reported PHM adherence and sociodemographic characteristics between people who used Twitter (subsequently rebranded X) and people who did not use Twitter.

**Methods:**

Repeated Canada-wide web-based surveys were conducted every 14 days from September 2020 to March 2022. Weighted proportions were calculated for descriptive variables. Using Bayesian logistic regression models, we investigated associations between Twitter use, as well as opinions in tweets, and self-reported adherence with mask wearing and vaccination.

**Results:**

Data from 40,230 respondents were analyzed. As self-reported, Twitter was used by 20.6% (95% CI 20.1%-21.2%) of Canadians, of whom 29.9% (95% CI 28.6%-31.3%) tweeted about COVID-19. The sociodemographic characteristics differed across categories of Twitter use and opinions. Overall, 11% (95% CI 10.6%-11.3%) of Canadians reported poor adherence to mask-wearing, and 10.8% (95% CI 10.4%-11.2%) to vaccination. Twitter users who tweeted about COVID-19 reported poorer adherence to mask wearing than nonusers, which was modified by the age of the respondents and their geographical region (odds ratio [OR] 0.79, 95% Bayesian credibility interval [BCI] 0.18-1.69 to OR 4.83, 95% BCI 3.13-6.86). The odds of poor adherence to vaccination of Twitter users who tweeted about COVID-19 were greater than those of nonusers (OR 1.76, 95% BCI 1.48-2.07). English- and French-speaking Twitter users who tweeted critically of PHMs were more likely (OR 4.07, 95% BCI 3.38-4.80 and OR 7.31, 95% BCI 4.26-11.03, respectively) to report poor adherence to mask wearing than non–Twitter users, and those who tweeted in support were less likely (OR 0.47, 95% BCI 0.31-0.64 and OR 0.96, 95% BCI 0.18-2.33, respectively) to report poor adherence to mask wearing than non–Twitter users. The OR of poor adherence to vaccination for those tweeting critically about PHMs and for those tweeting in support of PHMs were 4.10 (95% BCI 3.40-4.85) and 0.20 (95% BCI 0.10-0.32), respectively, compared to non–Twitter users.

**Conclusions:**

Opinions shared on Twitter can be useful to public health authorities, as they are associated with adherence to PHMs. However, the sociodemographics of social media users do not represent the general population, calling for caution when using tweets to assess general population-level behaviors.

## Introduction

### Background

Vaccination and nonpharmaceutical public health measures (PHMs) are used to reduce the spread of SARS-CoV-2 and minimize the occurrence of severe COVID-19, hospitalizations, and deaths. Vaccination provides some immunity against infection, reduces the severity of symptoms, and prevents death [1-3]. Nonpharmaceutical PHMs aim to minimize and prevent infectious contacts and include case detection and isolation, contact tracing and quarantine, travel restrictions, physical distancing, hand washing, and mask wearing. The effectiveness of vaccination against SARS-CoV-2 transmission was repetitively shown to be high, although heterogeneous for different populations and virus variants [4]. The reported effectiveness of nonpharmaceutical PHMs in lowering SARS-CoV-2 transmission is also variable [5,6]. Whether considering vaccination or nonpharmaceutical PHMs, effectiveness is influenced by the levels of coverage and adherence across the target population. During the pandemic, several methods have been used to measure adherence to PHMs. Public opinion research can measure the knowledge, attitudes, and behavior of respondents from panels of people who have agreed to participate [7]. Limitations of this approach include time- and resource-intensiveness, missing individuals reluctant to share their opinions, and errors from respondents who may not truthfully answer or misinterpret the meaning of survey questions.

Another potential source of adherence data is social media [8,9]. While the use of open-access social media data for public health has grown over the past 20 years, there are challenges. In particular, sociodemographic information of social media users is unavailable. Consequently, it is not possible to obtain a representative sample through sampling strategy or weights. Moreover, it is difficult to validate opinions and behaviors. For example, someone may tweet that they do not value COVID-19 vaccination, even if they are vaccinated.

### Objectives

The primary objective of this repeated cross-sectional study was to compare self-reported PHM adherence among people who tweeted about COVID-19 in the past 2 weeks, people who tweeted about something else in the past 2 weeks, and non–Twitter users. In addition, we aimed to compare the sociodemographic characteristics among these 3 groups, as well as among Twitter users, and to analyze reported PHM adherence according to the opinion regarding PHMs shared in their tweets.

## Methods

### Ethical Considerations

This project was approved by the Health Canada and the Public Health Agency of Canada Research Ethics Board (REB 2021-043P) and Comité d’éthique de la recherche en sciences et en santé at the University of Montreal (CERSES-20-056-D). Survey respondents were invited from the Angus Reid Forum, a large-scale national panel whose members receive small monetary compensation and occasional prize draws for completing surveys [10]. Consent was obtained through the Angus Reid platform, and participation to the panel and surveys was voluntary. Angus Reid reported participants could opt out at any time and refuse to answer any question. The data provided by Angus Reid were anonymized and deidentified. Although some information such as age and gender was provided, the identification of participants by a third party is very unlikely. Moreover, the data were kept in a secure server and were password protected.

### Recruitment

The repeated cross-sectional surveys were conducted every fortnight (cycles) from September 2020 to March 2022, except during the Canadian federal election (end of July 2021 to mid-November 2021). Moreover, 5 additional cycles were skipped due to financial restrictions (October 7, 2020; November 4, 2020; February 16, 2021; March 16, 2021; and March 30, 2021). While the Angus Reid Forum is not a probabilistic sample of the Canadian population, it includes enough panelists from basic demographic groups and regions to ensure that survey samples are similar to the adult Canadian population and weights are calculated according to demographic and regional data [10]. To minimize survey fatigue, forum respondents were removed from the pool of potential invitees for the 4 cycles after completing the survey. This means that some participants were repeated over cycles, but because respondents were asked to report behavior and social media use during the past 2 weeks, they were treated as unique respondents. The 27 cycles of survey data were divided into 6 periods representing different phases of the virus transmission and vaccination campaign in Canada (Table 1).

**Table 1:** Period division of the 40,230 respondents from 27 repeated surveys and the corresponding changes in the COVID-19 pandemic in Canada.

Period	Dates	Respondents, n (%)	Pandemic information
1	September 25, 2020, to December 16, 2020	7026 (17.46)	Start of the second wave
2	January 5, 2021, to March 4, 2021	6029 (14.99)	Peak of the second wave and start of the vaccination campaign targeting the older and most susceptible populations
3	April 14, 2021, to June 10, 2021	7550 (18.77)	Third wave, and Canadian adults became eligible for vaccination
4	June 25, 2021, to August 6, 2021	6003 (14.92)	In between waves (few cases occurred)
CFE^a,b^	September 20, 2021	0 (0)	Fourth wave
5	November 18, 2021, to December 23, 2021	6074 (15.1)	Inception of the fifth wave, largely driven by the Omicron variant
6	January 10, 2022, to March 8, 2022	7548 (18.76)	Peak of the fifth wave

^a^CFE: Canadian federal election.

^b^No surveys from the dissolution of Parliament (August 15, 2021) until after the unveiling of the new federal cabinet (October 26, 2021).

### Outcome Variables

Respondents were asked how often they wore a mask in public places in the previous fortnight. Adherence was dichotomized as poor (never, rarely, or sometimes) or good (every time or most of the time). Respondents were also asked whether they would be, or were already, vaccinated. The wording of the question changed according to the availability of vaccines (Multimedia Appendix 1). Adherence to vaccination was categorized into poor (would not be vaccinated) or good (was not sure, would be vaccinated, or had at least 1 dose of vaccine). The inclusion of respondents who were unsure about vaccination in the good adherence category was chosen as the vaccines were not approved at the beginning of the study and did not become available to everyone at the same time (Multimedia Appendix 1).

### Exposure Variables of Primary Interest

We were mainly interested in the association of 2 variables (referred to as exposure) with the adherence to mask wearing and vaccination (referred to as outcomes): the reported use of Twitter and related tweeted opinion. We used 2 approaches to measure Twitter use and the tweeted opinion about PHMs because the questions varied over the study period (Multimedia Appendix 2). Until August 2021 (periods 1-4), reported Twitter use in the previous fortnight was categorized as one of following: tweeting about COVID-19, not tweeting about COVID-19, or not using Twitter. In periods 5 and 6, question wording was changed such that no information on Twitter users not tweeting about COVID-19 was collected (Multimedia Appendix 2). For all periods, Twitter users who reported having posted about COVID-19 were asked to select all the specific COVID-19–related topics they had tweeted about in the previous fortnight (Multimedia Appendix 2), including PHMs and vaccination. Those selecting these options were asked whether they posted being in support, neutral, or critical of the PHMs. The 3-category opinion variable was combined with Twitter use to create a 5-category variable for regression analyses over all 6 periods: did not use Twitter or did not tweet about COVID-19, tweeted about COVID-19 but not about PHMs, tweeted in support of PHMs, tweeted about PHMs in a neutral manner, and tweeted critically about PHMs.

### Other Variables of Interest

Collected data also included survey date and respondent information, namely the province of residence, forward sortation area, age, gender, education level, occupation, household income, used official language, the country of origin, and ethnicity. We used the population size of the respondents’ residence by linking the forward sortation area to census data [11]. The daily containment and health index (CHI) for each province provided a measure for the implementation intensity of PHMs [5]. These data were obtained from a group at the Public Health Agency of Canada that used the methodology developed by the Oxford COVID-19 Government Response Tracker [12]. It was summarized as the median CHI per cycle per province and categorized into quartiles.

### Statistical Analyses

Statistical analyses were performed using R (version 4.2.2; R Foundation for Statistical Computing) [13]. For all analyses, data were considered cross-sectional due to the study design and the fact that Twitter use was reported over the 2 weeks before the survey. Descriptive statistics, including proportions with 95% CIs, were calculated for all periods using the weights provided by Angus Reid (*srvyr* package) [14]. Weighted proportions are presented in the *Results* section as proportion of Canadians. We considered the sociodemographic characteristics of participants to be similar to those of the overall Canadian population if the CI included the proportion reported by the National Census of Population 2021 [15]. Sociodemographic descriptive statistics by Twitter use categories were run for periods 1 to 4 only. We considered categories to be different when trends were outside the CI.

While there were no missing values in the survey answers, the option *prefer not to say* for income and ethnicity variables was considered as a missing value to minimize misclassification and removed from regression analyses (listwise deletion). Associations were estimated using Bayesian logistic regression models (*brms* package) [16]. Priors for regression coefficients were normal distributions centered on 0 with a precision of 1. Intercepts were normal distributions centered on 0 with a sigma of 1.

The modifying effect of selected covariables (region, period, age, gender, language, education, and CHI) on the association between the exposure of interest (Twitter use or combined tweeted opinions) and the 2 outcomes (vaccination and mask-wearing adherence) was assessed. Additional covariables were added to the model to adjust for confounding as follows: to avoid including collider variables, the variables of the minimal sufficient adjustment set identified in a directed acyclic graph (Multimedia Appendix 3) were added one at a time and kept for the final model if the beta coefficients changed by >10% [17]. Briefly, the directed acyclic graph included 10 covariables (sociodemographic variables, period, and CHI) and their expected association with Twitter use or opinions (exposures), PHMs’ adherence (outcome), and each other. Associations were based on previous findings about social media use [18,19], census information [11,15], and hypotheses from the research team. The final models were generated using 3 chains with a length of 3000, in which the first 1000 iterations were used as burn-in [20]. Convergence was monitored through the visual inspection of trace plots of variance components and density plots and by obtaining effective sample sizes. Goodness of fit was assessed using the correct classification rate and area under the curve [21]. Results are presented as odds ratio (OR) with 95% Bayesian credibility intervals (BCIs).

## Results

### Respondents

Survey data included 40,230 survey responses. Sociodemographic characteristics of the respondents varied slightly throughout the 6 periods (Multimedia Appendix 4). Table 2 compares the weighted distribution of respondents over the 6 periods to that of the Canadian census [15]. Several differences were noted, for example, for gender, household income, age, and the country of origin (Table 2).

**Table 2:** Weighted proportion of sociodemographic characteristics by Twitter use among 26,608 respondents to web-based surveys from September 2020 to mid-August 2021 and among all respondents (n=40,230; from September 2020 to March 2022)^a^.

Variable		Twitter use (periods 1-4^b^), weighted proportion (95% CI)				All respondents (periods 1-6^b^), weighted proportion (95% CI)		Census 2021 (%)
		Did not use (n=20,902)	Other tweets (n=4023)	COVID-19 tweets (n=1683)				
**Region**								
	British Columbia	13.3 (12.8-13.8)	12.9 (11.8-14.1)	10.6 (8.9-12.3)	13.1 (12.7-13.4)		13.5	
	Prairies^c^	17.9 (17.3-18.5)	17 (15.7-18.3)	19.5 (17.4-21.6)	17.9 (17.5-18.3)		18.2	
	Ontario	35.6 (34.9-36.3)	46.4 (44.6-48.1)	44.4 (41.6-47.0)	37.6 (37.2-38.2)		38.5	
	Quebec	26 (25.3-26.7)	15.3 (14.0-16.6)	20 (17.8-22.3)	24.1 (23.6-24.6)		23	
	Atlantic^d^	7.2 (6.8-7.6)	8.4 (7.4-9.3)	5.5 (4.3-6.8)	7.3 (7.0-7.6)		6.5	
**Population size**								
	Large (≥100,000)	70.7 (70.0-71.4)	76.2 (74.7-77.7)	77.6 (75.3-79.9)	71.5 (70.9-71.9)		NR^e^	
	Medium (30,000-99,999)	12.1 (11.6-12.6)	10.4 (9.3-11.4)	9.8 (8.2-11.4)	11.9 (11.6-12.3)		NR	
	Small (<30,000)	17.2 (16.7-17.8)	13.4 (12.2-14.6)	12.6 (10.8-14.4)	16.6 (16.2-17.0)		NR	
**Age (y)**								
	18-24	5.5 (5.1-5.8)	7.2 (6.3-8.1)	7.2 (5.9-8.6)	5.3 (5.1-5.6)		7.6^f^	
	25-34	22.4 (21.8-23.1)	21.8 (20.3-23.2)	24 (21.6-26.4)	22.9 (22.4-23.4)		17.1	
	35-44	16.2 (15.6-16.7)	18.4 (17.1-19.7)	21.8 (19.5-24.0)	16.7 (16.3-17.1)		17	
	45-54	16.8 (16.2-17.4)	21.1 (19.7-22.7)	20.9 (18.7-23.2)	17.9 (17.4-18.3)		16.1	
	55-64	18.1 (17.5-18.7)	17.2 (15.9-18.4)	12.1 (10.5-13.8)	17.2 (16.8-17.6)		18	
	65-74	16.1 (15.6-16.6)	11.7 (10.7-12.7)	11.7 (10.1-13.2)	15.6 (15.3-16.0)		14	
	≥75	4.9 (4.6-5.2)	2.6 (2.1-3.1)	2.3 (1.6-3.0)	4.4 (4.2-4.6)		10.2	
**Gender**								
	Men	46.8 (46.0-47.5)	52.1 (50.4-53.8)	53.4 (50.7-56.1)	48 (47.4-48.4)		49.3	
	Women	53.2 (52.5-54.0)	47.9 (46.2-49.6)	46.6 (43.9-49.3)	52 (51.5-52.6)		50.7	
	Other	—^g^	—	—	<0.1		NR	
**Education**								
	High school	34.8 (34.0-35.6)	29.4 (27.6-31.2)	31.6 (28.8-34.4)	33.8 (33.2-34.4)		32.9	
	College or trade school	33.3 (32.6-34.0)	31.7 (30.2-33.3)	30.7 (28.3-33.1)	32.9 (32.4-33.4)		34.3	
	University	31.9 (31.3-32.5)	38.9 (37.3-40.4)	37.7 (35.3-40.1)	33.3 (32.8-33.8)		32.9	
**Household income (CAD $)^h,i^**								
	<50,000	28.1 (27.4-28.9)	23.8 (22.2-25.4)	29.4 (26.7-32.0)	27.9 (27.4-28.4)		30.9	
	50,000-74,999	18.9 (18.2-19.5)	17.5 (16.1-18.9)	15.2 (13.2-17.2)	18.7 (18.3-19.2)		16.8^i^	
	75,000-99,999	17.6 (16.9-18.2)	16.2 (14.8-17.5)	17.3 (15.3-19.4)	17.2 (16.8-17.7)		20.6^j^	
	≥100,000	35.4 (34.7-36.2)	42.5 (40.8-44.3)	38.1 (35.4-40.8)	36.2 (35.6-36.7)		31.9	
**Official language**								
	English	79.7 (79.1-80.4)	89.9 (88.8-91.0)	84 (81.9-86.1)	81.4 (81.0-81.9)		NR	
	French	20.3 (19.6-20.9)	10.1 (9.0-11.2)	16 (13.9-18.1)	18.6 (18.1-19.0)		NR	
**Country of origin**								
	Canada	89.1 (88.7-89.5)	88.2 (87.2-89.3)	87.7 (86.0-89.4)	88.9 (88.6-89.2)		76.4	
	Abroad	10.9 (10.5-11.3)	11.8 (10.7-12.8)	12.3 (10.6-14.0)	11.1 (10.8-11.4)		23.6	
**Ethnicity^k^**								
	European ancestry	85.8 (85.3-86.3)	82.7 (81.4-84.0)	80.8 (78.7-82.9)	84.8 (84.4-85.2)		N/A^l^	
	Indigenous, First Nation, Inuit, or Metis	4.7 (4.3-5.0)	5.1 (4.3-6.0)	5.3 (4.1-6.5)	4.8 (4.6-5.1)		N/A	
	Other ethnic ancestries	9.5 (9.1-10.0)	12.1 (11.0-13.3)	13.9 (12.1-15.7)	10.4 (10.1-10.8)		N/A	

^a^The distribution of the Canadian population (census 2021) is also presented when available [15].

^b^Period 1: mid-September to the end of December 2020; period 2: January to March 2021; period 3: April to mid-June 2021; period 4: mid-June to mid-August 2021; period 5: mid-November to the end of December 2021; and period 6: January to March 2022.

^c^Alberta, Saskatchewan, and Manitoba.

^d^New Brunswick, Nova Scotia, Prince Edward Island, and Newfoundland and Labrador.

^e^NR: not reported.

^f^20 to 24 years of age in the 2021 census.

^g^Weighted estimates were 0 (0-0).

^h^Excluding the respondents who answered, “prefer not to say” (4229/40,230, 10.51%).

^i^According to the Bank of Canada, the annual conversion rates to US $ for the study period were 1.34 (2020), 1.25 (2021), and 1.30 (2022).

^j^CAD $50,000 to $69,999 and CAD $70,000 to $99,999 in the 2021 census.

^k^Excluding the respondents who answered, “Prefer not to say” (581/40,230, 1.44%).

^l^N/A: not available due to the reporting format (reporting combinations of ethnic origins, not the first reported one).

### Twitter Use and Tweeted Opinions About PHMs

During periods 1 to 4, 20.6% (95% CI 20.1%-21.2%) of Canadians reported using Twitter in the previous fortnight (Multimedia Appendix 4), of whom 29.9% (95% CI 28.6%-31.3%) tweeted about COVID-19 at least once. Table 2 shows the weighted distribution of respondents by the type of reported Twitter use, compared to that of the whole sampled population and to the census data. For example, compared to the overall study population or Canadian census, Twitter nonusers were overrepresented in Quebec but underrepresented in Ontario. Indeed, the proportion of Twitter users was lower in Quebec (14.3%, 95% CI 13.3%-15.3%) than in other provinces (British Columbia: 19.3%, 95% CI 17.8%-20.7%; Prairies: 20.5%, 95% CI 19.3%-21.8%; Ontario: 25%, 95% CI 24.1%-25.9%; and Atlantic: 21.4%, 95% CI 19.4%-23.4%). There was a greater proportion of Twitter users in large (22%, 95% CI 21.3%-22.6%) than in medium (18%, 95% CI 16.4%-19.5%) and small (16.5%, 95% CI 15.3%-17.8%) population size areas, among younger (aged 18 to 54 years; 23%, 95% CI 22.3%-23.8%) than among older (aged ≥55 years; 16.6%, 95% CI 15.8%-17.3%) age groups, in men (22.6%, 95% CI 21.8%-23.4%) than in women (18.8%, 95% CI 18.1%-19.6%), and in people with university education (23.9%, 95% CI 23.1%-24.7%) than in people with lower levels of education (high school: 18.3%, 95% CI 17.3%-19.4%; college: 19.7%, 95% CI 18.8%-20.5%). Moreover, there was a lower proportion of Twitter users of European ancestry (19.9%, 95% CI 19.4%-20.5%) than Twitter users of Indigenous, First Nation, Inuit, or Metis (22.4%, 95% CI 19.7%-25.1%) or of other ethnic ancestries (25.7%, 95% CI 23.9%-27.5%).

**Figure 1:**
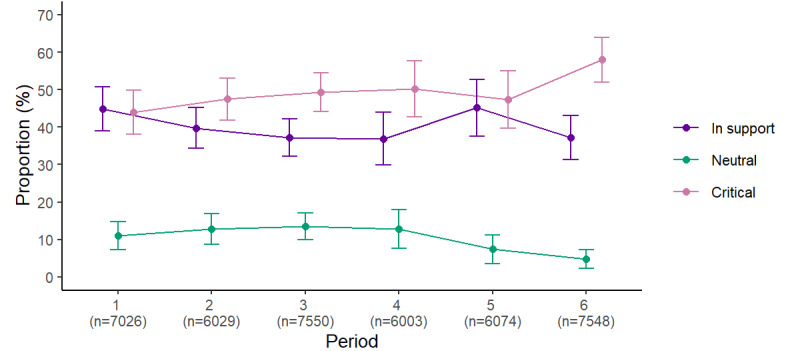
Weighted proportions (95% CIs) of respondents who tweeted in support, critically, or neutrally about public health measures from September 2020 to March 2022 (n=1840).

Of the Twitter users who tweeted about PHMs, 39.8% (95% CI 37.4%-42.3%) self-declared that their tweets were in support of PHMs, 49.4% (95% CI 46.9%-51.9%) self-declared that their tweets were critical, and 10.7% (95% CI 9.2%-12.3%) self-declared that their tweets were neutral. Opinions about PHMs varied over the study period, with a greater proportion of tweets supporting PHMs in period 1 and a greater proportion of critical tweets in period 6 (Figure 1). A greater proportion of Quebec, Ontario, and Prairies inhabitants reported tweeting critically about PHMs than those from the Atlantic provinces and British Columbia (Table 3). This was also the case for men, those with an annual household income >CAD $100,000 (according to the Bank of Canada, the annual conversion rate to US $ for the study period were 1.34 in 2020, 1.25 in 2021, and 1.30 in 2022), and those without a university degree (Table 3).

**Table 3:** Proportion of the sociodemographic characteristics of 1916 respondents to web-based surveys (September 2020 to March 2022) who tweeted in support of, neutrally about, and critically about public health measures.

Variable		In support (n=804), proportion (95% CI)	Neutral (n=194), proportion (95% CI)	Critical (n=918), proportion (95% CI)
**Region**				
	British Columbia	103 (47.7 (40.2-55.1)	26 (13.2 (7.5-18.9)	84 (39.1 (31.9-46.4)
	Prairies^a^	166 (32.1 (27.2-37.0)	33 (9.2 (5.8-12.6)	224 (58.7 (53.4-64.1)
	Ontario	377 (42.2 (38.6-45.8)	83 (9.7 (7.6-11.8)	422 (48.1 (44.5-51.7)
	Quebec	100 (33.5 (27.5-39.5)	39 (14.6 (9.9-19.3)	141 (51.9 (45.4-58.4)
	Atlantic^b^	58 (49.7 (39.9-59.5)	13 (9.5 (4.3-14.7)	47 (40.8 (31.1-50.4)
**Population size**				
	Large (≥100,000)	635 (40.3 (37.6-43.1)	156 (11.2 (9.3-13.0)	719 (48.5 (45.7-51.3)
	Medium (30,000-99,999)	80 (39.5 (31.6-47.4)	19 (10.1 (5.4-14.9)	80 (50.4 (42.1-58.6)
	Small (<30,000)	89 (37.6 (30.7-44.4)	18 (8.4 (4.4-12.4)	118 (54 (46.9-61.1)
**Age (y)**				
	18-24	32 (35.8 (25.5-46.1)	17 (20.6 (11.7-29.5)	40 (43.6 (33.1-54.3)
	25-34	164 (40.7 (35.2-46.3)	52 (13.2 (9.3-17.0)	179 (46.1 (40.5-51.7)
	35-44	172 (37.2 (32.1-42.4)	35 (8.8 (5.6-11.9)	209 (54 (48.6-59.4)
	45-54	181 (38.4 (33.3-43.5)	37 (8.4 (5.5-11.2)	224 (53.2 (47.9-58.5)
	55-64	128 (40.1 (34.0-46.3)	29 (12.4 (7.7-17.2)	144 (47.5 (41.1-53.7)
	65-74	106 (44.7 (38.1-51.2)	19 (7.8 (4.4-11.3)	108 (47.5 (40.9-54.1)
	≥75	21 (52.9 (37.2-68.6)	5 (13.5 (2.3-24.6)	14 (33.6 (18.9-48.4)
**Gender**				
	Men	420 (36.8 (33.6-40.0)	112 (10.8 (8.7-12.9)	538 (52.4 (49.1-55.8)
	Women	384 (43.5 (39.8-47.3)	82 (10.7 (8.3-13.1)	380 (45.8 (42.0-49.6)
**Education**				
	High school	113 (35.1 (29.5-40.8)	43 (12.8 (8.9-16.7)	167 (52.1 (46.2-58.0)
	College or trade school	229 (37.1 (33.1-41.1)	51 (9.1 (6.6-11.6)	312 (53.9 (49.7-58.0)
	University	462 (45.5 (42.3-48.7)	100 (10.6 (8.6-12.7)	439 (43.9 (40.7-47.1)
**Household income (CAD $)^c^**				
	<50,000	164 (44.3 (38.6-50.0)	50 (13.5 (9.7-17.4)	157 (42.2 (36.6-47.8)
	50,000-74,999	117 (42.8 (36.4-49.3)	22 (10.7 (6.0-15.4)	134 (46.5 (40.0-53.0)
	75,000-99,999	134 (42.2 (36.2-48.3)	36 (11.7 (7.8-15.7)	132 (46 (39.8-52.3)
	≥100,000	329 (34.6 (31.2-38.1)	72 (8.7 (6.6-10.8)	429 (56.7 (53.0-60.4)
**Official language**				
	English	734 (41.2 (38.6-43.8)	168 (10 (8.4-11.6)	806 (48.8 (46.2-51.5)
	French	70 (30.9 (24.2-37.6)	26 (15.8 (9.7-21.9)	112 (53.3 (45.8-60.8)
**Country of origin**				
	Canada	701 (39.5 (36.9-42.1)	156 (9.8 (8.2-11.4)	826 (50.7 (48.0-53.4)
	Abroad	103 (42.4 (35.5-49.4)	38 (18 (12.2-23.8)	92 (39.6 (32.7-46.4)
**Ethnicity^d^**				
	European ancestry	658 (39 (36.4-41.7)	143 (9.8 (8.1-11.5)	790 (51.2 (48.5-54.0)
	Indigenous, First Nation, Inuit, or Metis	45 (48.3 (36.4-60.1)	7 (9.4 (2.4-16.5)	30 (42.3 (30.3-54.3)
	Other ethnic ancestry	93 (42.2 (35.0-49.2)	41 (18.8 (13.2-24.5)	85 (39 (32.0-46.0)

^a^Alberta, Saskatchewan, and Manitoba.

^b^New Brunswick, Nova Scotia, Prince Edward Island, and Newfoundland and Labrador.

^c^Excluding the respondents who answered, “prefer not to say” (135/1776, 7.6%) According to the Bank of Canada, the annual conversion rates to US $ for the study period were 1.34 (2020), 1.25 (2021), and 1.30 (2022).

^d^Excluding the respondents who answered, “prefer not to say” (24/1892, 1.27%).

### Adherence to PHMs

Overall, 11% (95% CI 10.6%-11.3%) of Canadians reported poor mask-wearing adherence, which varied by period between 5.9% and 16.3%, with the highest value reported in period 4 (Multimedia Appendix 5). Mask-wearing adherence also varied by sociodemographic characteristics, with French-speaking respondents and women wearing a mask more frequently than English-speaking respondents and men, respectively. Mask-wearing frequency increased with age and education level, and decreased with income (Multimedia Appendix 6). Similarly, 10.8% (95% CI 10.4%-11.2%) of Canadians reported poor adherence to vaccination, which also varied by period. The proportion of people who reported poor adherence was 15.1% (95% CI 13.9%-16.3%) in period 1, which decreased to a minimum of 7.4% (95% CI 5.7%-9.2%) in period 4 before increasing again to 9.8% (95% CI 9.0%-10.6%) in period 6 (Multimedia Appendix 5). Vaccination adherence also varied by sociodemographic characteristics, with a greater proportion of Canadians who would not be vaccinated in the Prairies than in other regions, among men than women, among those aged between 18 and 54 years than among the older groups, and in urban areas than in rural areas. The proportion of Canadians in favor of vaccination increased with education level and household income (Multimedia Appendix 7). During periods 2 and 3 (not measured in other periods), 91.1% (95% CI 90.3%-92.0%) of Canadians who were planning to receive vaccination said they would also continue to follow other preventive health measures, including mask wearing.

### Association Among Twitter Use, Tweeted Opinion, and Adherence to PHMs

The proportion of Canadians who reported poor adherence to PHMs differed by Twitter use and tweeted opinion. Overall, 19% (95% CI 16.4%-21.6%) of those who tweeted about COVID-19 had poor adherence to vaccination, and the proportion of those who had poor adherence to mask wearing varied by geographical region and age (Tables 4 and 5). The highest proportion of respondents with poor adherence to mask wearing and vaccination was in the subset who tweeted critically about PHMs (29.6% to 33.1%), while the lowest proportion was in the subset who tweeted in support of PHMs (2.2% to 5.2%; Tables 6 and 7).

In comparing non–Twitter users to participants who tweeted about COVID-19, the adjusted OR of poor adherence to mask wearing ranged from 0.79 (95% BCI 0.18-1.69) to 4.83 (95% BCI 3.13-6.86; Table 4). Geographical region and age were effect modifiers: the association was not observed in the Prairies and the Atlantic provinces, and the strength of the association decreased with age. In comparing non–Twitter users to those who tweeted about something else, the adjusted OR of poor adherence to mask wearing ranged from 0.27 (95% BCI 0.04-0.67) to 1.02 (95% BCI 0.59-1.51; Table 4). The adjusted OR of poor vaccination adherence was 1.76 (95% BCI 1.48 to 2.07) for participants who tweeted about COVID-19 and 0.67 (95% BCI 0.57 to 0.78) for those who tweeted about something else compared to non–Twitter users (Table 5).

The adjusted ORs of poor adherence to mask wearing for participants tweeting critically about PHMs were 4.07 (95% BCI 3.38-4.80) for English-speaking participants and 7.31 (95% BCI 4.26-11.03) for French-speaking participants, compared to non–Twitter users and those who did not tweet about COVID-19 (Table 6). For participants tweeting in support of PHMs compared to non–Twitter users and those who did not tweet about COVID-19, the adjusted ORs were 0.47 (95% BCI 0.31-0.64) and 0.96 (95% BCI 0.18-2.33) for English- and French-speaking participants, respectively (Table 6). The adjusted ORs of poor vaccination adherence for those tweeting critically about PHMs and for those tweeting in support of PHMs were 4.10 (95% BCI 3.40-4.85) and 0.20 (95% BCI 0.10-0.32), respectively, compared to non–Twitter users or those not tweeting about COVID-19 (Table 7).

**Table 4:** Weighted proportions of respondents with poor mask-wearing adherence by Twitter use, and effect modifier categories, between September 2020 and November 2021 (n=21,054)^a^.

Variable	Poor adherence, weighted proportion (95% CI)	Unadjusted OR^b^ (95% BCI^c^)	Adjusted OR^d^ (95% BCI)
Did not use Twitter in British Columbia	14.8 (13.2-16.4)	Reference	Reference
Tweeted about something else in British Columbia	10 (6.3-13.8)	0.66 (0.47-0.88)	0.53 (0.34-0.73)
Tweeted about COVID-19 in British Columbia	30.2 (21.5-38.9)	2.25 (1.52-3.13)	2.00 (1.27-2.95)
Did not use Twitter in the Prairies^e^	18.7 (17.1-20.3)	Reference	Reference
Tweeted about something else in the Prairies^e^	13.7 (10.6-16.7)	0.75 (0.58-0.95)	0.57 (0.41-0.77)
Tweeted about COVID-19 in the Prairies^e^	23.8 (18.1-29.4)	1.29 (0.95-1.69)	1.26 (0.85-1.73)
Did not use Twitter in Ontario	8.2 (7.5-9.0)	Reference	Reference
Tweeted about something else in Ontario	6.7 (5.3-8.0)	0.80 (0.64-0.99)	0.66 (0.49-0.87)
Tweeted about COVID-19 in Ontario	18 (14.8-21.2)	2.35 (1.86-2.90)	2.35 (1.71-3.00)
Did not use Twitter in Quebec	5 (4.3-5.8)	Reference	Reference
Tweeted about something else in Quebec	6.2 (3.8-8.5)	1.23 (0.81-1.73)	0.89 (0.53-1.31)
Tweeted about COVID-19 in Quebec	21.8 (16.2-27.3)	5.13 (3.54-6.83)	4.83 (3.13-6.86)
Did not use Twitter in the Atlantic provinces^f^	6.3 (4.8-7.9)	Reference	Reference
Tweeted about something else in the Atlantic provinces^f^	6.4 (3.2-9.6)	0.96 (0.50-1.53)	0.89 (0.45-1.44)
Tweeted about COVID-19 in the Atlantic provinces^f^	4 (0-8.8)	0.88 (0.23-1.88)	0.79 (0.18-1.69)
Did not use Twitter for age 18 to 24 years	10.4 (8.3-12.4)	Reference	Reference
Tweeted about something else for age 18 to 24 years	7.4 (4.0-10.8)	0.72 (0.45-1.07)	0.75 (0.43-1.13)
Tweeted about COVID-19 for age 18 to 24 years	29.5 (20.2-38.8)	2.92 (1.81-4.33)	3.15 (1.82-4.84)
Did not use Twitter for age 25 to 34 years	11.6 (10.4-12.9)	Reference	Reference
Tweeted about something else for age 25 to 34 years	9.6 (7.1-12.1)	0.89 (0.68-1.16)	0.93 (0.65-1.22)
Tweeted about COVID-19 for age 25 to 34 years	26.1 (20.7-31.5)	2.94 (2.21-3.77)	2.75 (1.90-3.77)
Did not use Twitter for age 35 to 44 years	11 (9.7-12.3)	Reference	Reference
Tweeted about something else for age 35 to 44 years	8.7 (6.3-11.2)	0.79 (0.57-1.03)	0.74 (0.52-0.99)
Tweeted about COVID-19 for age 35 to 44 years	22 (16.6-27.5)	2.01 (1.47-2.68)	1.84 (1.20-2.56)
Did not use Twitter for age 45 to 54 years	10.5 (9.3-11.8)	Reference	Reference
Tweeted about something else for age 45 to 54 years	8.3 (5.7-10.9)	0.69 (0.48-0.90)	0.71 (0.48-0.97)
Tweeted about COVID-19 for age 45 to 54 years	17.1 (12.4-21.9)	1.74 (1.24-2.40)	1.69 (1.10-2.41)
Did not use Twitter for age 55 to 64 years	9.8 (8.6-10.9)	Reference	Reference
Tweeted about something else for age 55 to 64 years	7.3 (5.1-9.5)	0.80 (0.56-1.06)	0.78 (0.53-1.09)
Tweeted about COVID-19 for age 55 to 64 years	13.9 (8.3-19.5)	1.48 (0.88-2.16)	1.40 (0.78-2.18)
Did not use Twitter for age 65 to 74 years	6.9 (6.0-7.8)	Reference	Reference
Tweeted about something else for age 65 to 74 years	7.3 (4.7-9.9)	1.05 (0.64-1.49)	1.02 (0.59-1.51)
Tweeted about COVID-19 for age 65 to 74 years	15.1 (9.8-20.4)	2.44 (1.53-3.55)	2.14 (1.18-3.29)
Did not use Twitter for age ≥75 years	8.6 (6.8-10.5)	Reference	Reference
Tweeted about something else for age ≥75 years	4.4 (0.1-8.7)	0.51 (0.14-1.04)	0.27 (0.04-0.67)
Tweeted about COVID-19 for age ≥75 years	5.9 (0-13.8)	1.03 (0.22-2.32)	0.98 (0.16-2.30)

^a^Odds ratio and 95% Bayesian credibility interval values were obtained from Bayesian logistic regression models built using repeated cross-sectional data from surveys of Canadians.

^b^OR: odds ratio.

^c^BCI: Bayesian credibility interval.

^d^Adjusted for gender, education level, language, income, population size, and period.

^e^Alberta, Saskatchewan, and Manitoba.

^f^New Brunswick, Nova Scotia, Prince Edward Island, and Newfoundland and Labrador.

**Table 5:** Weighted proportions of respondents with poor vaccination adherence by Twitter use between September 2020 and November 2021 (n=18,036)^a^.

Variable	Poor adherence, weighted proportion (95% CI)	Unadjusted OR^b^ (95% BCI^c^)	Adjusted OR^d^ (95% BCI)
Did not use Twitter	12.2 (11.5-12.8)	Reference	Reference
Tweeted about something else	8.2 (6.9-9.4)	0.64 (0.54-0.73)	0.67 (0.57-0.78)
Tweeted about COVID-19	19 (16.4-21.6)	1.67 (1.40-1.94)	1.76 (1.48-2.07)

^a^Odds ratio and 95% Bayesian credibility interval values were obtained from Bayesian logistic regression models built using repeated cross-sectional data from surveys of Canadians.

^b^OR: odds ratio.

^c^BCI: Bayesian credibility interval.

^d^Adjusted for age, education level, population size, and period.

**Table 6:** Weighted proportions of respondents with poor mask-wearing adherence by shared opinions on Twitter, and effect modifier categories, between September 2020 and March 2022 (n=33,296)^a^.

Variable	Poor adherence, weighted proportion (95% CI)	Unadjusted OR^b^ (95% BCI^c^)	Adjusted OR^d^ (95% BCI)
Did not tweet about COVID-19 or use Twitter for English-speaking respondents	11.6 (11.2-12.1)	Reference	Reference
Did not tweet about PHMs^e^ for English-speaking respondents	13.9 (11.6-16.2)	1.26 (1.05-1.49)	1.03 (0.84-1.24)
Tweeted in support of PHMs for English-speaking respondents	5.0 (3.3-6.7)	0.47 (0.32-0.63)	0.47 (0.31-0.64)
Tweeted neutrally about PHMs for English-speaking respondents	10.9 (5.5-16.3)	1.01 (0.56-1.52)	1.00 (0.53-1.54)
Tweeted critically about PHMs for English-speaking respondents	33.1 (29.3-36.9)	3.90 (3.30-4.54)	4.07 (3.38-4.80)
Did not tweet about COVID-19 or use Twitter for French-speaking respondents	5.3 (4.6-5.9)	Reference	Reference
Did not tweet about PHMs for French-speaking respondents	14.4 (8.7-20.2)	2.82 (1.69-4.16)	2.69 (1.59-4.06)
Tweeted in support of PHMs for French-speaking respondents	5.2 (0-10.5)	0.96 (0.19-2.10)	0.96 (0.18-2.33)
Tweeted neutrally about PHMs for French-speaking respondents	26.3 (2.5-50.0)	2.84 (0.65-6.76)	3.13 (0.60-7.63)
Tweeted critically about PHMs for French-speaking respondents	29.6 (20.0-39.2)	8.43 (5.26-12.31)	7.31 (4.26-11.03)

^a^Odds ratio and 95% Bayesian credibility interval values were obtained from Bayesian logistic regression models built using repeated cross-sectional data from surveys of Canadians.

^b^OR: odds ratio.

^c^BCI: Bayesian credibility interval.

^d^Adjusted for gender, age, education level, income, population size, containment and health index, and period.

^e^PHM: public health measure.

**Table 7:** Weighted proportions of respondents with poor vaccination adherence by shared opinions on Twitter between September 2020 and March 2022 (n=28,343)^a^.

Variable	Poor adherence, weighted proportion (95% CI)	Unadjusted OR^b^ (95% BCI^c^)	Adjusted OR^d^ (95% BCI)
Did not tweet about COVID-19 or use Twitter	10.5 (10.0-10.9)	Reference	Reference
Did not tweet about PHMs^e^	10.8 (8.7-12.9)	0.98 (0.79-1.18)	1.12 (0.90-1.35)
Tweeted in support of PHMs	2.2 (0.7-3.6)	0.19 (0.10-0.30)	0.20 (0.10-0.32)
Tweeted neutrally about PHMs	18.9 (11.1-26.7)	1.75 (1.06-2.66)	1.62 (0.91-2.44)
Tweeted critically about PHMs	31.7 (27.9-35.6)	4.07 (3.47-4.77)	4.10 (3.40-4.85)

^a^Odds ratio and 95% Bayesian credibility interval values were obtained from Bayesian logistic regression models built using repeated cross-sectional data from surveys of Canadians.

^b^OR: odds ratio.

^c^BCI: Bayesian credibility interval.

^d^Adjusted for age, income, containment health index, and period.

^e^PHM: public health measure.

## Discussion

### Principal Findings

This is the first individual-level repeated cross-sectional study to measure the magnitude of the association, in the context of COVID-19, of self-reported Twitter use and tweeted content with self-reported mask-wearing and vaccination behaviors. Previous studies were all susceptible to ecological bias and confounding linked to the sociodemographic characteristics of social media users [8,9]. Our study lasted nearly 2 years and used web-based survey data from participants weighted to be representative of the Canadian population. Our results underscore the sociodemographic differences among types of Twitter users and non–Twitter users. Similar to other study findings [18,19], more Twitter users were younger men and living in large cities with a higher income or level of education compared to nonusers. Furthermore, our study provides evidence that people who tweet critically about PHMs are also less likely to comply with these measures.

Among all study participants, there was an overall 89% adherence to both mask wearing and vaccination. Among participants tweeting about COVID-19, however, the adherence to mask wearing was between 70% and 96%, and the adherence to vaccination was 81%. This is somewhat comparable to what was found in a worldwide Twitter poll where vaccination intention in February 2021 was 83% [22]. Canadians who tweeted critically about PHMs and those who tweeted in support of PHMs differed drastically in their adherence to mask wearing (67% to 70%, and 95%, respectively) and vaccination (68% and 98%, respectively). These results suggest that sentiments shared on Twitter are linked to adherence to PHMs but that these estimates are not representative of overall adherence. It is possible these discrepancies would be observed for other social media platforms, but this would need to be confirmed. For example, correlations were found between the sentiment of tweets extracted using an algorithm, announcement of nonpharmaceutical interventions from governmental entities, and reported compliance [23]. However, our results highlight the importance of considering sociodemographic and behavioral discrepancies in the general population, given the types of social media use and nonuse, when using health intelligence from social media for health surveillance applications [24].

The association between tweeting about COVID-19 as well as opinions about PHMs and poor adherence to PHMs was confirmed in multivariable analyses adjusting for sociodemographic confounders. Although we did not explore the underlying explanation for the association between Twitter use and PHM adherence, our findings align with a Canadian study that found an association between social media exposure and having more misperceptions and less social distancing adherence, compared to exposure to traditional news media [25]. Other studies have reported a negative association between believing in misinformation and adherence to PHMs [26-28], although not specific to social media exposure. Therefore, our findings may reflect greater misperceptions among some Twitter users [25].

The association between Twitter use and adherence to mask wearing was not uniform across age or geographical regions. The association between Twitter use and adherence to mask wearing weakened with age, except among those aged 65 to 74 years. From the beginning of the pandemic, this age group was a highly susceptible population and generally reported high adherence to PHMs [29,30], which could explain this finding. Regional differences in the association between Twitter use and adherence to mask wearing reflected stronger associations between mask wearing and Twitter use in Quebec and no associations in the Atlantic provinces [31]. There was a greater adherence to mask wearing in both these regions than in the other regions, but the Twitter user population differed. In the Atlantic provinces, the proportion of adherence to PHMs was high across Twitter users and nonusers. This could be due to the aggressive implementation of PHMs early in the pandemic, which allowed a return to economic and social activities [32]. There was a smaller proportion of Twitter users in Quebec, but those who tweeted had generally low support for PHMs. Perhaps the few Twitter users in Quebec were more prone to use this medium to voice their discontent with PHMs than those in other regions. A major difference in Quebec is that French is the official language and is the primary language spoken. While 79% of the Quebec population speaks French at home, this proportion ranged from <1% (British Columbia, Alberta, Saskatchewan, and Newfoundland and Labrador) to 28% (New Brunswick) in other provinces [15]. While geographical region was not an effect modifier for the model assessing the association between adherence to mask wearing and opinions shared on Twitter, language was. There are French-speaking communities across the country, but this interaction is likely driven by differences in Quebec. These results point to the need to account for regional and cultural differences when using social media data for estimating populations’ behaviors.

### Social Media and Real-Time Monitoring of PHM Adherence

Open-access social media are among the most timely and voluminous digital information sources for public health surveillance to infer population-level health-related behaviors. Application program interfaces (APIs) enable efficient collection of high volumes of posts. Some APIs allow targeted sample collection, such as sample collection by content (eg, keywords and hashtags), languages, and regions. Associated metadata from collected social media publications can include a user identifier, time stamp of publication, and republication status. Some users make the location of publication or residence public. The publications and associated metadata can then be used to derive geolocated indicators to measure real- or near real–time changes in health-related behaviors. Possible indicators include time-series variables for the volume of publications, volume of publications about a topic, or sentiment of publications or topics. Monitoring social media for trends in PHM adherence could improve agility in adapting PHMs and communication strategies to encourage adherence. Likewise, real-time signals of low adherence could provide advanced warning for areas at a higher risk of disease emergence or re-emergence.

Public health institutes could capitalize on using social media for health intelligence through event-based surveillance systems and other tools that scan open-access data from the internet for evidence of public health threats [33,34]. However, there are ethical and practical challenges to using social media for public health surveillance, which are constantly evolving. People who post publicly may not be aware that their data are being collected or used for purposes beyond their intent, and this raises ethical, legal, and privacy considerations [35]. In addition, the open access to data through APIs can be discontinued at any moment, which happened with Twitter following the ownership change in 2022. Representative sampling from the population of interest is also difficult. Platforms may be used differently over time and according to social demographic factors [36]. Furthermore, social media contain misinformation and disinformation about health, which can influence health-related behaviors [37].

### Limitations and Biases

The surveyed population was slightly different from the Canadian population [15], with an overrepresentation of younger people with a high income. This could have driven part of the sociodemographic characteristics we observed for Twitter users and overestimated the proportion of Twitter users in Canada [18,19]. However, it likely had a limited impact on the adjusted associations.

Classification errors were also possible. First, we asked respondents to self-report their adherence to PHMs; answers could have been influenced by social desirability bias [38]. It is possible that Canadians who tend to tweet in support of PHMs would be more susceptible to social desirability bias, while those tweeting critically might overstate their nonadherent behaviors. Hence, we cannot discount the possibility of differential bias when estimating the type of Twitter use. This is less likely to have happened for the measurement of Twitter use. Second, the classification used for good and poor adherence could have resulted in errors: we determined that respondents with poor adherence to vaccination included only respondents who would definitely not get vaccinated and classified respondents who were unsure as having good adherence. We used this approach because of the change in vaccine approval and availability throughout the study period [31] and to allow for the aggregation of answer choices that also changed (Multimedia Appendices 1 and 2). We classified good adherence to mask wearing as wearing a mask in public “most of the time” or “all the time.” The classification bias resulting from these decisions would likely underestimate the proportion of Canadians with poor adherence, but the direction or magnitude of this bias on association measures is difficult to predict.

### Conclusions

We found that Twitter users who tweeted about COVID-19 had different sociodemographic characteristics and reported lower adherence to PHMs than users who did not tweet about COVID-19 and nonusers. Moreover, the lowest adherence was among Twitter users who tweeted critically about PHMs. Furthermore, the proportion of people tweeting in support of (or critically about) PHMs was not a good indicator of adherence to PHMs in the general population. Study trends were relatively stable over the 2-year study period, supporting the utility of using indicators of health-related behavior from social media over a limited multiyear period. However, because sociodemographic information from social media users is usually unavailable, our results call for caution in using social media information to estimate support for and adherence to PHMs in the general population. Future research should determine whether adjustments using sociodemographic characteristics could mitigate this, improving the ability to predict behavior and adherence and, ultimately, to characterize or forecast trends in disease transmission.

## Appendix

Multimedia Appendix 1 Questions and answer options for vaccination status for a repeated cross-sectional study assessing Twitter use and adherence to public health measures.

Multimedia Appendix 2 Questions and answer options for Twitter use for a repeated cross-sectional study assessing Twitter use and adherence to public health measures.

Multimedia Appendix 3 Directed acyclic graph for the association between Twitter use and adherence to 2 public health measures: mask wearing and vaccination.

Multimedia Appendix 4 Weighted proportions of the sociodemographic characteristics of 40,230 respondents to web-based surveys over 6 periods.

Multimedia Appendix 5 Weighted proportions of mask-wearing and vaccination adherence of Canadian respondents from September 2020 to March 2022.

Multimedia Appendix 6 Weighted proportions of mask-wearing frequency in public places for 37,222 respondents to web-based surveys from September 2020 to March 2022 by period and sociodemographic characteristics.

Multimedia Appendix 7 Weighted proportions of the vaccination status of 31,666 respondents to web-based surveys from September 2020 to March 2022 by period and sociodemographic characteristics.

## Abbreviations

API: application program interface
BCI: Bayesian credibility interval
CHI: containment and health index
OR: odds ratio
PHM: public health measure
