# Supplementary material for: Linking Opinions Shared on Social Media About COVID-19 Public Health Measures to Adherence: Repeated Cross-Sectional Surveys of Twitter Use in Canada
Source: J Med Internet Res. 2024 Aug 13;26:e51325. doi: 10.2196/51325 (PMC11350311; doi:10.2196/51325)
Supplement: Multimedia Appendix 1 [file jmir_v26i1e51325_app1.docx]

**Multimedia Appendix 1.** Questions and answer options for vaccination status for a repeated cross-sectional study assessing Twitter use and adherence to public health measures.

|  | **Version 1** | **Version 2** | **Version 3** | **Version 4** | **Version 5** |
| --- | --- | --- | --- | --- | --- |
|  |  |  |  |  |  |
| Dates | Nov. 2020 to Jan. 2021 | Feb. and Mar. 2021 | Apr. to mid-May 2021 | Mid-May to Aug. 2021 | Nov. 2021 to Feb. 2022 |
| Vaccine availability | Vaccine in development, announcement of upcoming availability on Dec. 14, 2020 | First doses available to health care personnel and elderly (varied by province) | First doses available to 50 years old and older in most provinces | First doses available to all adults in all provinces from the end of May, second doses available to 50+ | Booster available to all adults by the end of Dec. in all provinces |
| Vaccine status question | Would you be vaccinated against COVID-19 if a vaccine approved by Health Canada becomes available for Canadians? | Will you be vaccinated against COVID-19 when you become eligible to receive the vaccine? | Will you be vaccinated against COVID-19 when you become eligible to receive the vaccine? | Would you be vaccinated against COVID-19 with a vaccine approved by Health Canada when you are eligible? | Would you be vaccinated against COVID-19 with a vaccine approved by Health Canada when you are eligible (including if you are eligible for a booster)? |
| Answer options | 1. Yes 2. No 3. Not sure | 1. Yes 2. No 3. Not sure | 1. Yes 2. No 3. Not sure 4. I have already been vaccinated | 1. Yes 2. No 3. Not sure 4. I have already been vaccinated once 5. I have already been vaccinated twice | 1. Yes 2. No 3. Not sure 4. I have already been vaccinated with one dose 5. I have already been vaccinated with two doses 6. I have already been vaccinated with two doses and received a booster |
