# Supplementary material for: Linking Opinions Shared on Social Media About COVID-19 Public Health Measures to Adherence: Repeated Cross-Sectional Surveys of Twitter Use in Canada
Source: J Med Internet Res. 2024 Aug 13;26:e51325. doi: 10.2196/51325 (PMC11350311; doi:10.2196/51325)
Supplement: Multimedia Appendix 2 [file jmir_v26i1e51325_app2.docx]

**Multimedia Appendix 2.** Questions and answer options for Twitter use for a repeated cross-sectional study assessing Twitter use and adherence to public health measures.

|  | **Version 1** | **Version 2** |
| --- | --- | --- |
|  |  |  |
| Dates | Sep. 2020 to Aug. 2021 | Nov. 2021 to Feb. 2022 |
| Selection question | None | Which social media platforms do you use to publicly post content about COVID-19 (i.e. photos, videos, text-based messages, etc.)?  * If Twitter was selected, the respondent had access to the next question. |
| Twitter use question | “In the last 2 weeks, have you posted on Twitter (or tweeted) about any of the  following related to COVID-19?” | |
| Answer options | 1. Symptoms you or someone you know have experienced 2. Having gone through (or about to go through) the testing process for you or someone you know 3. Test results for you or someone you know | 1. Symptoms you or someone you know have experienced 2. Having gone through (or about to go through) the testing process for you or someone you know 3. Test results for you or someone you know |
|  | 1. Your perceptions about public health measures that are in place in your region (e.g. masking, social distancing, lockdown, isolation when sick, quarantine when exposed) | 1. Your perceptions about public health measures that are in place in your region (e.g. masking, social distancing, lockdown, isolation when sick, quarantine when exposed) 2. Your perceptions about the recommendation for you to receive the vaccine to reduce the risk of COVID-19 3. Your perceptions about the recommendation for your children (5-11 years old), if you’re a parent, to receive the vaccine to reduce the risk of COVID-19 |
|  | 1. I have posted on Twitter in the last 2 weeks but not about this | n.e. |
|  | n.e. | 1. None of the above |
| n.e. There was no equivalent answer choice between versions 1 and 2. | | |
