# Supplementary figures and images for: Linking Opinions Shared on Social Media About COVID-19 Public Health Measures to Adherence: Repeated Cross-Sectional Surveys of Twitter Use in Canada
Source: J Med Internet Res. 2024 Aug 13;26:e51325. doi: 10.2196/51325 (PMC11350311; doi:10.2196/51325)

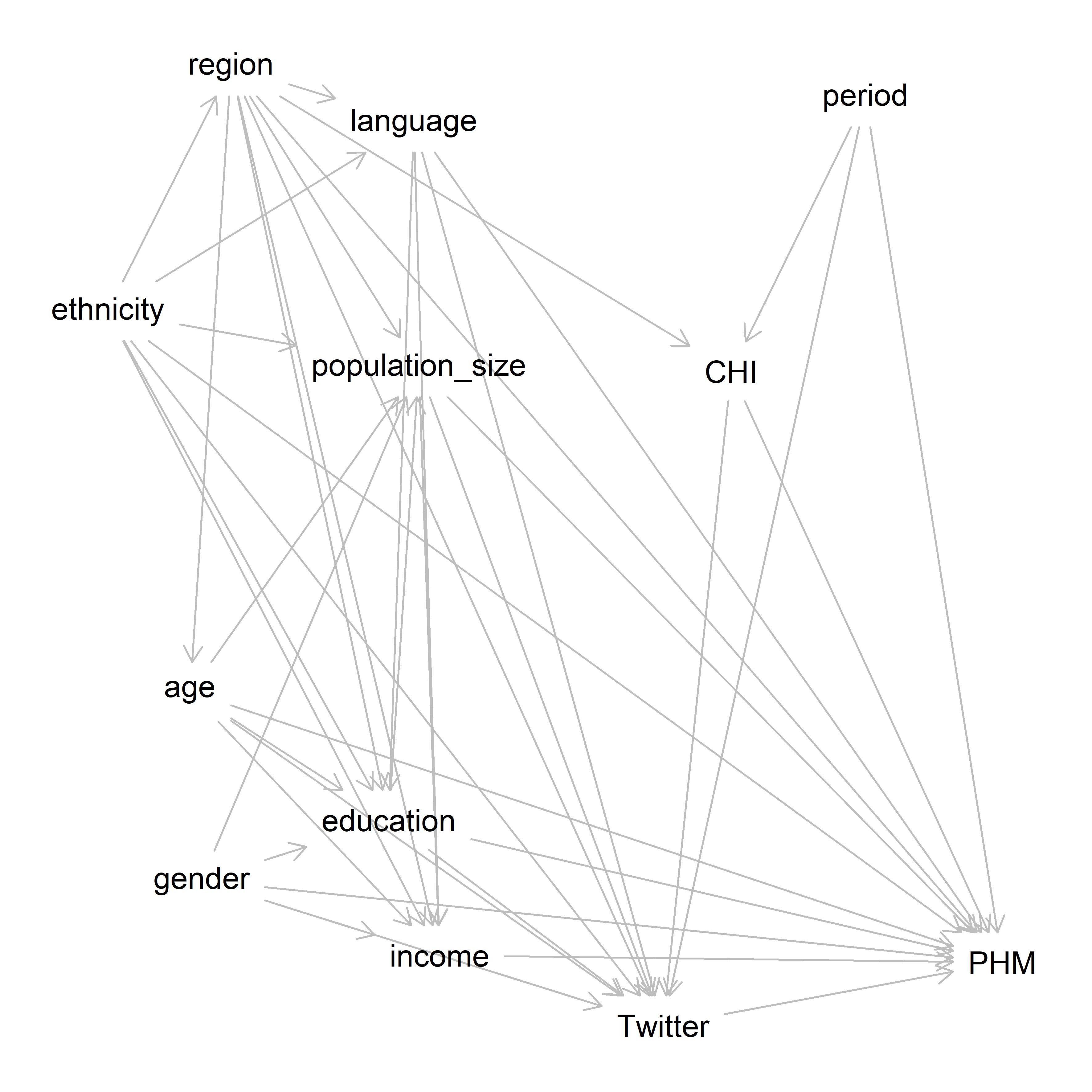

Supplement: Multimedia Appendix 3 [file jmir_v26i1e51325_app3.png]

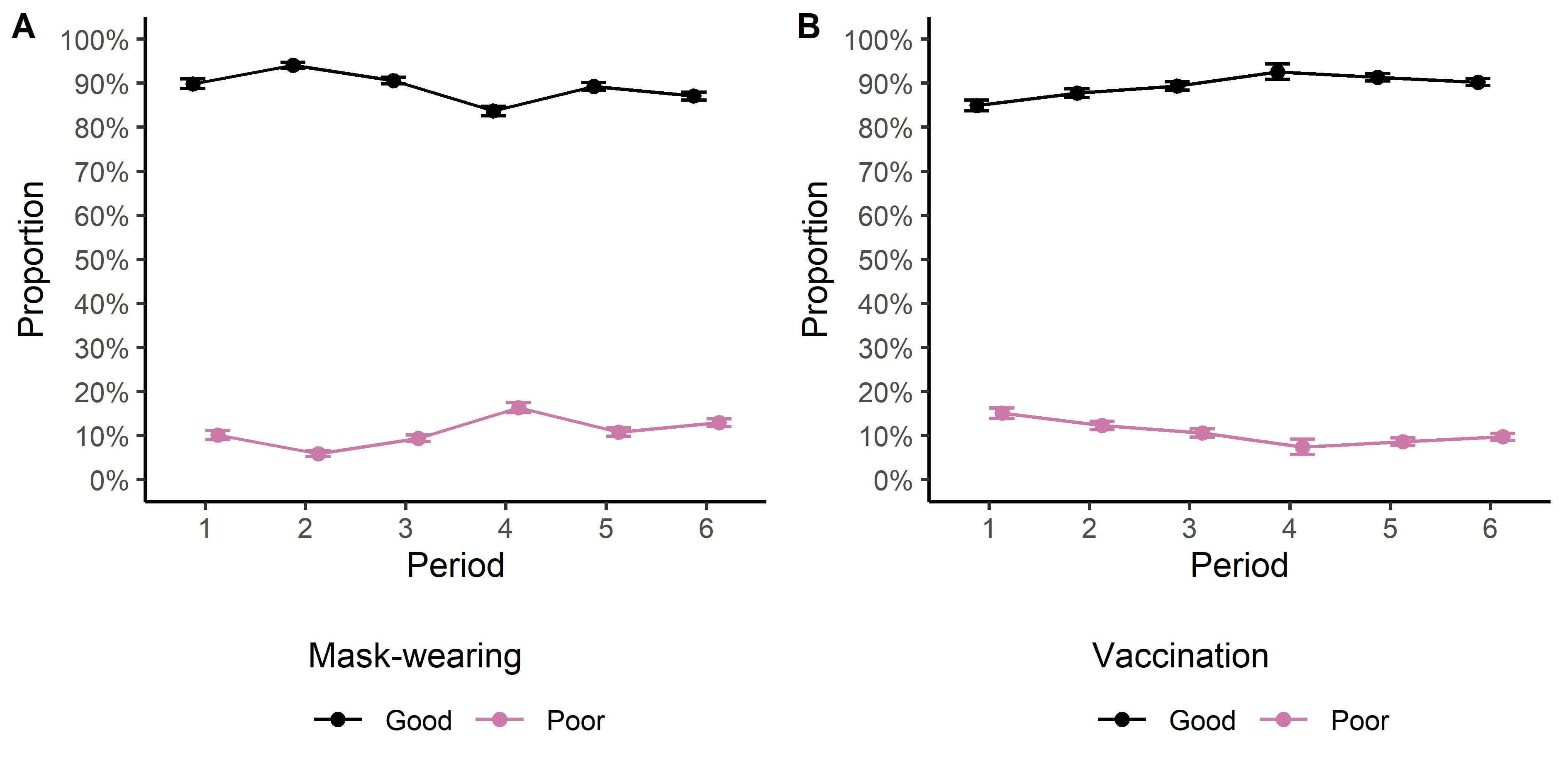

Supplement: Multimedia Appendix 5 [file jmir_v26i1e51325_app5.png]
