# Supplementary material for: Linking Opinions Shared on Social Media About COVID-19 Public Health Measures to Adherence: Repeated Cross-Sectional Surveys of Twitter Use in Canada
Source: J Med Internet Res. 2024 Aug 13;26:e51325. doi: 10.2196/51325 (PMC11350311; doi:10.2196/51325)
Supplement: Multimedia Appendix 6 [file jmir_v26i1e51325_app6.docx]

**Multimedia Appendix 6.** Weighted proportions of mask-wearing frequency in public places for 37,222 respondents to web-based surveys from September 2020 to March 2022 by period and sociodemographic characteristics.

|  |  | Mask-wearing frequency in public places | | |
| --- | --- | --- | --- | --- |
|  | n | Low (never,  rarely, sometimes) | Intermediate (most of the time) | High (all the time) |
|  |  |  |  |  |
| **Period** |  |  |  |  |
| 1: September to December 2020 | 4,018 | 10.1% (9.1; 11.2) | 13.0% (11.8; 14.1) | 76.9% (75.4; 78.3) |
| 2: January to March 2021 | 6,029 | 5.9% (5.2; 6.6) | 7.8% (7.1; 8.6) | 86.3% (85.3; 87.2) |
| 3: April to mid-June 2021 | 7,550 | 9.4% (8.6; 10.1) | 10.7% (9.9; 11.5) | 79.9% (78.9; 81.0) |
| 4: mid-June to August 2021 | 6,003 | 16.3% (15.2; 17.4) | 14.8% (13.8; 15.8) | 68.9% (67.5; 70.2) |
| 5: November to December 2021 | 6,074 | 10.8% (9.9; 11.7) | 11.9% (11.0; 12.8) | 77.3% (76.1; 78.5) |
| 6: January to March 2022 | 7,548 | 12.9% (12.1; 13.8) | 11.5% (10.7; 12.3) | 75.6% (74.5; 76.7) |
| **Region** |  |  |  |  |
| British Columbia | 5,080 | 14.6% (13.4; 15.8) | 16.8% (15.7; 18.0) | 68.6% (67.1; 70.0) |
| Prairies | 6,819 | 18.5% (17.5; 19.6) | 12.7% (11.8; 13.6) | 68.8% (67.5; 70.1) |
| Ontario | 14,341 | 9.8% (9.2; 10.3) | 11.3% (10.8; 11.9) | 78.9% (78.1; 79.6) |
| Québec | 8,430 | 6.3% (5.7; 6.9) | 8.7% (8.0; 9.4) | 85.0% (84.1; 85.8) |
| Atlantic | 2,552 | 7.5% (6.3; 8.7) | 8.9% (7.7; 10.1) | 83.6% (82.0; 85.2) |
| **Population size** |  |  |  |  |
| Large (≥ 100,000) | 27,025 | 9.7% (9.3; 10.1) | 11.7% (11.3; 12.1) | 78.6% (78.0; 79.2) |
| Medium (30,000 to 99,999) | 4,324 | 14.2% (13.1; 15.4) | 11.4% (10.3; 12.4) | 74.4% (72.9; 75.9) |
| Small (< 30,000) | 5,850 | 14.0% (13.0; 15.0) | 10.7% (9.9; 11.6) | 75.3% (74.1; 76.5) |
| **Age** |  |  |  |  |
| 18 to 24 | 1,860 | 12.6% (11.0; 14.2) | 15.2% (13.5; 16.9) | 72.2% (70.1; 74.3) |
| 25 to 34 | 7,903 | 13.7% (12.8; 14.6) | 14.3% (13.4; 15.2) | 72.0% (70.9; 73.2) |
| 35 to 44 | 6,387 | 12.5% (11.5; 13.4) | 12.3% (11.4; 13.2) | 75.2% (74.0; 76.4) |
| 45 to 54 | 6,324 | 11.6% (10.7; 12.5) | 11.4% (10.5; 12.3) | 77.0% (75.8; 78.2) |
| 55 to 64 | 6,832 | 9.3% (8.5; 10.1) | 9.0% (8.2; 9.7) | 81.7% (80.7; 82.8) |
| 65 to 74 | 6,194 | 7.0% (6.3; 7.6) | 8.7% (8.0; 9.5) | 84.3% (83.4; 85.2) |
| 75 + | 1,722 | 7.2% (5.9; 8.5) | 9.3% (7.9; 10.7) | 83.5% (81.7; 85.3) |
| **Gender** |  |  |  |  |
| Men | 17,861 | 14.7% (14.1; 15.3) | 14.4% (13.9; 15.0) | 70.9% (70.1; 71.6) |
| Women | 19,356 | 7.5% (7.1; 8.0) | 8.8% (8.3; 9.2) | 83.7% (83.1; 84.3) |
| Other | 5 | - | - | - |
| **Education** |  |  |  |  |
| High school | 8,374 | 12.9% (12.1; 13.7) | 10.9% (10.1; 11.6) | 76.2% (75.2; 77.2) |
| College or trade school | 12,037 | 11.8% (11.2; 12.4) | 11.9% (11.3; 12.5) | 76.3% (75.4; 77.0) |
| University | 16,811 | 8.2% (7.7; 8.6) | 11.7% (11.2; 12.2) | 80.2% (79.5; 80.8) |
| **Household income^a^** |  |  |  |  |
| Less than $50,000 | 8,399 | 10.6% (9.9; 11.4) | 10.9% (10.2; 11.7) | 78.5% (77.5; 79.5) |
| $50,000 to 74,999 | 6,072 | 10.5% (9.6; 11.4) | 11.3% (10.4; 12.1) | 78.2% (77.0; 79.4) |
| $75,000 to 99,999 | 5,761 | 11.1% (10.1; 12.0) | 11.5% (10.6; 12.4) | 77.4% (76.2; 78.7) |
| $100,000 or more | 13,087 | 11.7% (11.0; 12.3) | 12.7% (12.0; 13.3) | 75.6% (74.8; 76.5) |
| **Official language** |  |  |  |  |
| English | 30,953 | 12.1% (11.7; 12.5) | 12.3% (11.9; 12.7) | 75.6% (75.1; 76.2) |
| French | 6,269 | 6.0% (5.3; 6.7) | 8.1% (7.3; 8.8) | 85.9% (84.9; 86.9) |
| **Country of origin** |  |  |  |  |
| Canada | 32,688 | 11.0% (10.6; 11.4) | 11.4% (11.0; 11.8) | 77.6% (77.1; 78.1) |
| Abroad | 4,534 | 11.0% (9.9; 12.0) | 12.0% (11.0; 13.1) | 77.0% (75.6; 78.3) |
| **Ethnicity^b^** |  |  |  |  |
| Indigenous, First Nation, Inuit or Metis | 1,590 | 11.3% (9.5; 13.1) | 10.9% (9.2; 12.7) | 77.8% (75.4; 80.1) |
| European ancestry | 31,060 | 11.1% (10.7; 11.5) | 11.3% (10.9; 11.7) | 77.6% (77.1; 78.1) |
| Other ethnic ancestry | 4,037 | 9.7% (8.7; 10.8) | 13.6% (12.5; 14.8) | 76.7% (75.2; 78.1) |
| ^a^ Excluding the respondents who answered “Prefer not to say” (n = 3,903; 10.5%)  ^b^ Excluding the respondents who answered “Prefer not to say” (n = 535; 1.4%) | | | | |
