# Supplementary material for: Linking Opinions Shared on Social Media About COVID-19 Public Health Measures to Adherence: Repeated Cross-Sectional Surveys of Twitter Use in Canada
Source: J Med Internet Res. 2024 Aug 13;26:e51325. doi: 10.2196/51325 (PMC11350311; doi:10.2196/51325)
Supplement: Multimedia Appendix 7 [file jmir_v26i1e51325_app7.docx]

**Multimedia Appendix 7.** Weighted proportions of the vaccination status of 31,666 respondents to web-based surveys from September 2020 to March 2022 by period and sociodemographic characteristics.

|  |  | Vaccination status | | |
| --- | --- | --- | --- | --- |
|  | n | Would not be vaccinated | Was not sure if would be vaccinated | Would be or was vaccinated |
|  |  |  |  |  |
| **Period** |  |  |  |  |
| 1: September to December 2020 | 4,518 | 15.1% (13.9; 16.3) | 20.8% (19.5; 22.1) | 64.1% (62.6; 65.7) |
| 2: January to March 2021 | 6,029 | 12.3% (11.3; 13.3) | 15.6% (14.6; 16.7) | 72.1% (70.8; 73.4) |
| 3: April to mid-June 2021 | 6,037 | 10.6% (9.7; 11.6) | 8.0% (7.2; 8.9) | 81.4% (80.2; 82.5) |
| 4: mid-June to August 2021 | 1,460 | 7.4% (6.7; 9.2) | 4.0% (2.9; 5.2) | 88.6% (86.5; 90.6) |
| 5: November to December 2021 | 6,074 | 8.7% (7.8; 9.5) | 2.7% (2.2; 3.1) | 88.6% (87.8; 89.6) |
| 6: January to March 2022 | 7,548 | 9.8% (9.0; 10.6) | 2.0% (1.6; 2.4) | 88.2% (87.4; 89.1) |
| **Region** |  |  |  |  |
| British Columbia | 4,335 | 10.2% (9.1; 11.3) | 7.9% (7.0; 8.8) | 81.9% (80.6; 83.3) |
| Prairies | 5,799 | 16.2% (15.1; 17.3) | 9.5% (8.7; 10.4) | 74.3% (72.9; 75.6) |
| Ontario | 12,192 | 9.6% (9.0; 10.3) | 8.6% (8.0; 9.2) | 81.8% (81.0; 82.6) |
| Québec | 7,164 | 9.9% (9.1; 10.7) | 9.0% (8.2; 9.7) | 81.1% (80.1; 82.2) |
| Atlantic | 2,176 | 8.2% (6.9; 9.5) | 7.3% (6.0; 8.5) | 84.5% (82.8; 86.2) |
| **Population size** |  |  |  |  |
| Large (≥ 100,000) | 22,877 | 9.0% (8.5; 9.4) | 8.3% (7.9; 8.7) | 82.7% (82.1; 83.3) |
| Medium (30,000 to 99,999) | 3,741 | 15.1% (13.8; 16.4) | 7.9% (7.0; 8.9) | 77.0% (75.4; 78.5) |
| Small (< 30,000) | 5,027 | 15.7% (14.5; 16.8) | 10.6% (9.7; 11.6) | 73.7% (72.3; 75.1) |
| **Age** |  |  |  |  |
| 18 to 24 | 1,564 | 11.3% (9.6; 13.0) | 10.9% (9.3; 12.5) | 77.8% (75.6; 80.0) |
| 25 to 34 | 6,722 | 13.5% (12.5; 14.5) | 9.4% (8.6; 10.2) | 77.1% (75.9; 78.3) |
| 35 to 44 | 5,411 | 12.8% (11.7; 13.9) | 9.8% (8.8; 10.7) | 77.4% (76.1; 78.7) |
| 45 to 54 | 5,401 | 12.3% (11.3; 13.4) | 10.2% (9.3; 11.1) | 77.5% (76.1; 78.7) |
| 55 to 64 | 5,798 | 9.6% (8.7; 10.5) | 8.9% (8.0; 9.7) | 81.5% (80.4; 82.7) |
| 65 to 74 | 5,311 | 5.9% (5.2; 6.5) | 4.9% (4.3; 5.4) | 89.2% (88.4; 90.2) |
| 75 + | 1,459 | 5.6% (4.4; 6.9) | 4.4% (3.3; 5.4) | 90.0% (88.4; 91.6) |
| **Gender** |  |  |  |  |
| Men | 15,193 | 12.0% (11.4; 12.6) | 8.3% (7.8; 8.8) | 79.7% (79.0; 80.5) |
| Women | 16,468 | 9.8% (9.2; 10.3) | 9.0% (8.5; 9.5) | 81.2% (80.5; 81.9) |
| Other | 5 | - | - | - |
| **Education** |  |  |  |  |
| High school | 7,221 | 15.5% (14.6; 16.5) | 11.3% (10.5; 12.1) | 73.2% (72.0; 74.3) |
| College or trade school | 10,377 | 11.7% (11.0; 12.3) | 8.9% (8.3; 9.5) | 79.4% (78.6; 80.2) |
| University | 14,068 | 5.2% (4.8; 5.6) | 5.7% (5.3; 6.1) | 89.1% (88.6; 89.7) |
| **Household income^a^** |  |  |  |  |
| Less than $50,000 | 7,283 | 13.1% (12.2; 14.0) | 10.3% (9.5; 11.1) | 76.6% (75.4; 77.7) |
| $50,000 to 74,999 | 5,107 | 11.0% (10.0; 12.0) | 8.4% (7.5; 9.2) | 80.6% (79.4; 81.9) |
| $75,000 to 99,999 | 4,910 | 10.4% (9.4; 11.4) | 8.0% (7.1; 8.9) | 81.6% (80.3; 82.8) |
| $100,000 or more | 11,043 | 8.9% (8.3; 9.6) | 7.2% (6.7; 7.8) | 83.9% (83.0; 84.7) |
| **Official language** |  |  |  |  |
| English | 26,268 | 10.9% (10.4; 11.3) | 8.5% (8.1; 8.9) | 80.6% (80.0; 81.1) |
| French | 5,398 | 10.6% (9.7; 11.6) | 9.2% (8.3; 10.1) | 80.2% (78.9; 81.4) |
| **Country of origin** |  |  |  |  |
| Canada | 27,801 | 11.1% (10.7; 11.6) | 8.5% (8.1; 8.9) | 80.4% (79.8; 80.9) |
| Abroad | 3,865 | 8.6% (7.6; 9.6) | 9.9% (8.9; 11.0) | 81.5% (80.1; 82.8) |
| **Ethnicity^b^** |  |  |  |  |
| Indigenous, First Nation, Inuit or Metis | 1,369 | 15.6% (13.4; 17.7) | 10.4% (8.5; 12.2) | 74.0% (71.4; 76.7) |
| European ancestry | 26,415 | 10.7% (10.3; 11.2) | 8.1% (7.7; 8.5) | 81.2% (80.6; 81.7) |
| Other ethnic ancestry | 3,428 | 9.0% (7.9; 10.1) | 11.7% (10.4; 12.9) | 79.3% (77.8; 80.9) |
| ^a^ Excluding the respondents who answered “Prefer not to say” (n = 3,323; 10.5%)  ^b^ Excluding the respondents who answered “Prefer not to say” (n = 454; 1.4%) | | | | |
